# Tumor-Infiltrating T Cells From Clear Cell Renal Cell Carcinoma Patients Recognize Neoepitopes Derived From Point and Frameshift Mutations

**DOI:** 10.3389/fimmu.2020.00373

**Published:** 2020-03-12

**Authors:** Ulla Kring Hansen, Sofie Ramskov, Anne-Mette Bjerregaard, Annie Borch, Rikke Andersen, Arianna Draghi, Marco Donia, Amalie Kai Bentzen, Andrea Marion Marquard, Zoltan Szallasi, Aron Charles Eklund, Inge Marie Svane, Sine Reker Hadrup

**Affiliations:** ^1^Department of Health Technology, Technical University of Denmark, Lyngby, Denmark; ^2^Center for Cancer Immune Therapy, Copenhagen University Hospital, Copenhagen, Denmark; ^3^Danish Cancer Society Research Center, Copenhagen, Denmark; ^4^Clinical Microbiomics A/S, Copenhagen, Denmark

**Keywords:** renal cell carcinoma, neoepitopes, neoantigens, frameshift mutations, T cell screening

## Abstract

Mutation-derived neoantigens are important targets for T cell-mediated reactivity toward tumors and, due to their unique tumor expression, an attractive target for immunotherapy. Neoepitope-specific T cells have been detected across a number of solid cancers with high mutational burden tumors, but neoepitopes have been mostly selected from single nucleotide variations (SNVs), and little focus has been given to neoepitopes derived from in-frame and frameshift indels, which might be equally important and potentially highly immunogenic. Clear cell renal cell carcinomas (ccRCCs) are medium-range mutational burden tumors with a high pan-cancer proportion of frameshift mutations. In this study, the mutational landscape of tumors from six RCC patients was analyzed by whole-exome sequencing (WES) of DNA from tumor fragments (TFs), autologous tumor cell lines (TCLs), and tumor-infiltrating lymphocytes (TILs, germline reference). Neopeptides were predicted using MuPeXI, and patient-specific peptide–MHC (pMHC) libraries were created for all neopeptides with a rank score < 2 for binding to the patient's HLAs. T cell recognition toward neoepitopes in TILs was evaluated using the *high-throughput* technology of DNA barcode-labeled pMHC multimers. The patient-specific libraries consisted of, on average, 258 putative neopeptides (range, 103–397, *n* = 6). In four patients, WES was performed on two different sources (TF and TCL), whereas in two patients, WES was performed only on TF. Most of the peptides were predicted from both sources. However, a fraction was predicted from one source only. Among the total predicted neopeptides, 16% were derived from frameshift indels. T cell recognition of 52 neoepitopes was detected across all patients (range, 4–18, *n* = 6) and spanning two to five HLA restrictions per patient. On average, 21% of the recognized neoepitopes were derived from frameshift indels (range, 0–43%, *n* = 6). Thus, frameshift indels are equally represented in the pool of immunogenic neoepitopes as SNV-derived neoepitopes. This suggests the importance of a broad neopeptide prediction strategy covering multiple sources of tumor material, and including different genetic alterations. This study, for the first time, describes the T cell recognition of frameshift-derived neoepitopes in RCC and determines their immunogenic profile.

## Introduction

Tumor neoantigens are important targets for the immune system to mediate tumor control. Tumor-specific mutations give rise to altered proteins that are processed into short peptides. These are presented at the cell surface in the context of major histocompatibility complex (MHC) molecules, where they serve as targets for cytotoxic T cell killing of the tumor (1). Compared to shared tumor antigens, which can be expressed at low levels in healthy tissue, neoantigens have the advantage of being uniquely expressed in the tumor. Also, there is less T cell tolerance toward neoantigens since the T cell repertoire has not been negatively selected based on these sequences (2). Therefore, neoantigens are attractive targets for immunotherapy. Untargeted therapies, such as immune checkpoint inhibitors and adoptive T cell transfer with tumor infiltrating lymphocytes, have been shown to increase neoantigen reactive T cells, and the clinical response correlates with the mutational burden and predicted number of neoantigens (3–5). Neoantigens have also been directly targeted in personalized therapies by adoptive transfer of specifically expanded T cells (6, 7) and in personalized neoepitope vaccines (8, 9). The challenge for these strategies is, however, to determine which neoepitopes to preferentially target in each patient. Neoantigen reactive T cells have been detected across a number of solid cancers with high mutational burdens, such as melanoma and non-small cell lung cancer (10–12). The described neopeptides have, however, mainly been derived from single nucleotide variations (SNVs) with less focus on in-frame and frameshift indels, mutation types that are likely to be immunogenic based on their large sequence variance to the germline DNA. Even though the total number of frameshift indels are lower than SNVs, they have been shown to give rise to three times as many predicted high-affinity (IC_50_ < 50 nM) neoantigens per mutation compared to SNVs and are highly enriched for mutant-specific binding (i.e., neopeptides for which the wild-type peptide is not predicted to bind the HLA) (13). Hence, this mutation type is potentially highly relevant as a tumor neoantigen target (14, 15).

Clear cell renal cell carcinomas (ccRCCs) are medium-range mutational burden tumors that present with the highest pan-cancer proportion of frameshift indels (13, 16). ccRCCs appear to be immune sensitive, as suggested by high levels of T cells infiltrating the tumor site (17), and clinical benefit can be achieved using cytokine-based immunotherapies with interferon-α and high-dose interleukin 2 (18, 19) and checkpoint inhibitors (20, 21). Nevertheless, the tumor microenvironment of ccRCCs is characterized as highly immunosuppressive (22), which is reflected by the poor functional quality of T cell responses observed, with implications for adoptive cell therapy (23).

To our knowledge, as yet, no reports have described the neoantigens recognized by T cells in ccRCC and investigated the contribution of frameshift indels to T cell recognition of neoantigens. Such investigation is critical for using neoantigens as therapeutic targets and biomarkers of relevance to immunotherapy in this cancer type. For that reason, we evaluated the T cell recognition of neopeptides predicted from SNVs, in-frame, and frameshift indels in six ccRCC patients previously described in (23). The prediction was performed with WES from two sources of tumor material (TCL and TF) to include all potential neopeptides in our screenings.

## Materials and Methods

### Patient and Healthy Donor Samples

Healthy donor samples were collected by approval of the local Scientific Ethics Committee, with donor written informed consent obtained according to the Declaration of Helsinki. Healthy donor blood samples were obtained from the blood bank at Rigshospitalet, Copenhagen, Denmark. All samples were obtained anonymously. Peripheral blood mononuclear cells (PBMCs) from healthy donors were obtained from whole blood by density centrifugation on Lymphoprep (Axis-Shield PoC, cat# 1114544) in Leucosep tubes (Greiner Bio-One, cat# 227288) and cryopreserved at −150°C in fetal calf serum (FCS, Gibco, cat#10500064) + 10% dimethyl sulfoxide (DMSO, Sigma-Aldrich, cat#C6164).

Tumor-infiltrating lymphocytes (TILs), tumor fragments (TFs), and tumor cell lines (TCLs) from ccRCC patients were obtained at the Department of Oncology and Center for Cancer Immune Therapy, Copenhagen University Hospital, Denmark, under approval by the Ethics Committee of the Capital Region of Denmark and the Danish Data Protection Agency. Young TIL cultures were obtained from resected tumor lesions from individuals with ccRCC with a Fuhrman grade between 1 and 3 (23). Tumor lesions were resected following surgical removal, and TFs were cultured individually in complete medium [RPMI1640 + GlutaMAX^TM^ (Gibco, cat#61870010) with 10% human serum (Sigma-Aldrich, cat#H3667), 100 U/ml penicillin (P/S, Sigma-Aldrich, cat#P0781), 100 μg/ml streptomycin (P/S, Sigma-Aldrich, cat#P0781), 1.25 μg/ml fungizone (Bristol-Myers Squibb), and 6,000 U/ml IL-2 (Proleukin, Novartis, cat#200-02)] at 37°C and 5% CO_2_, allowing TILs to migrate into the medium. TILs were expanded to reach >50 × 10^6^ total cells originating from ~48 individual fragments, which had expanded to confluent growth in 2 ml wells and eliminated adherent tumor cells (average of ~2 × 10^6^ cells per well from each TF). TIL cultures were further expanded using a standard rapid expansion protocol (REP) as previously described (24). Briefly, TILs were stimulated with 30 ng/ml anti-CD3 antibody (OKT-3, Ortho Biotech) and 6,000 U/ml IL-2 in the presence of irradiated (40 Gy) allogeneic feeder cells (healthy donor PBMCs) at a feeder/TIL ratio of 200:1. Initially, TILs were rapidly expanded in a 1:1 mix of complete medium and REP medium [AIM-V (Invitrogen) + 10% human serum, 1.25 μg/ml fungizone, and 6,000 U/ml IL-2], but after 7 days, complete medium and serum were removed stepwise from the culture by adding REP medium without serum to maintain cell densities around 1–2 × 10^6^ cells/ml. TIL cultures were cryopreserved at −150°C in human serum + 10% DMSO.

### DNA and RNA Extraction and Sequencing Preparation

DNA and RNA were extracted and purified from TCLs, TFs, and TILs (germline DNA reference) using the AllPrep DNA/RNA Mini kit (Qiagen, cat#80204), with the addition of DNase during RNA purification (Qiagen, 79254). Next, DNA/RNA concentrations were analyzed by NanoDrop (Thermo Fischer Technologies), and RNA RIN values were analyzed by 2100 Bioanalyzer (Agilent Technologies). DNA whole-exome and RNA sequencing (RNAseq) were performed at the DTU Multi Assay Core (DMAC).

### Next-Generation Sequencing Data Processing

Raw FASTQ files from whole WES and RNAseq were analyzed in the following manner. First, both data sets were pre-processed for quality using Trim Galore version 0.4.0 (25), which combines the functions of Cutadapt (26) and FastQC 0.11.2 (27): trimming the reads below an average Phred score of 20 (default value), cutting out standard adaptors such as those from Illumina, and running FastQC to evaluate data quality. Variant calling was performed following the Genome Analysis Toolkit (GATK) best practice guidelines for somatic variant detection (28). Reads were aligned to the human genome (GRCh38) using the Burrows-Wheeler Aligner (29) version 0.7.10 with default mem options and with a reading group provided for each sample for compatibility with the following steps. Duplicate reads were marked using Picard-tools version 2.6.0 MarkDuplicates. Indel realignment and base recalibration were performed with GATK version 3.3.0 to reduce false-positive variant calls. SNV and indel calls were made using GATKs build in a version of MuTect2 (30) designed to call variants, both SNVs and indels, from matched tumor and normal samples. Kallisto 0.42.1 (31) was used to determine the gene expression in transcript per million (TPM) from RNAseq data.

### Neopeptide Prediction

The VCF output files from GATK's MuTect2 was given as input to the neopeptide predictor MuPeXI version 1.1 (32) together with RNAseq expression values obtained from Kallisto. HLA alleles of each patient were inferred from the WES data using OptiType version 1.0 (33) with default settings after filtering the reads aligning to the HLA region with RazerS version 3.4.0 (34). Identified mutations from TFs and TCLs were used to predict 9, 10, and 11 amino acid peptides, sorted according to the eluted ligand percentile rank (EL% Rank) score of the mutated neopeptides using netMHCpan 2.8 (35). All neopeptides with a rank score < 2 were selected for peptide synthesis, giving a total of 1,545 neopeptides across all six patients. Additionally, the tumor mutational burden of non-synonymous mutations was determined from the MuPeXI output logfile summarizing peptides originating from missense variant mutations, in-frame insertions, and deletions, together with frameshift mutations. Mutation types were determined by Ensembl's variant effect predictor as a dependency of MuPeXI. The neopeptide prediction has, prior to publication, been reanalyzed with MuPeXI 1.2.0 using netMHCpan 4.0 (36).

### Peptides

All selected mutation derived and virus control peptides were purchased from Pepscan (Pepscan Presto BV, Lelystad, Netherlands) and dissolved to 10 mM in DMSO.

### MHC Monomer Production and Generation of Specific Peptide–MHC Complexes

The production of MHC monomers was performed as previously described (37, 38). In brief, the heavy chains of the included HLA types and human β_2_ microglobulin (β_2_m) light chain were expressed in bacterial Bl21 (DE3) pLysS strain (Novagen, cat#69451) and purified as inclusion bodies. After solubilization, heavy-chain and β2m light-chain complexes were folded using a UV-sensitive ligand (39, 40), biotinylated with BirA biotin-protein ligase standard reaction kit (Avidity, 318 LLC-Aurora, Colorado), and purified using size-exclusion column (Waters, BioSuite125, 13 μm SEC 21.5 × 300 mm) HPLC (Waters 2489). Specific peptide–MHC (pMHC) complexes were generated by UV-induced peptide exchange (37, 39).

### Detection of pMHC Specific T Cells by DNA Barcode-Labeled Multimers

Patient-specific libraries of predicted neopeptides and virus control peptides (size range 114–415 peptides) were generated as previously described (41). Briefly, the pMHC complexes generated above were coupled to a phycoerythrin (PE)- and DNA barcode-labeled dextran backbone. Hence, a specific peptide was given a unique DNA barcode together with a PE-fluorescent label. ccRCC patient TILs and healthy donor PBMCs were stained with an up-concentrated pool of all multimers in the presence of 50 nM dasatinib, followed by staining with a 5× antibody mix composed of CD8-BV510 (BD 563256, clone RPA-T8) or -BV480 (BD, cat#566121, clone RPA-T8), dump channel antibodies [CD4-FITC (BD, cat#345768), CD14-FITC (BD, cat#345784), CD19-FITC (BD, cat#345776), CD40-FITC (Serotech, cat#MCA1590F), and CD16-FITC (BD, cat#335035)], and a dead cell marker (LIVE/DEAD Fixable Near-IR; Invitrogen, cat#L10119). Multimer binding T cells were sorted as lymphocytes, single, live, CD8^+^, FITC^−^, and PE^+^ and pelleted by centrifugation. DNA barcodes were amplified from the isolated cells and from a stored aliquot of multimer pool (diluted 50,000× in the final PCR reaction, used as a baseline). PCR products were purified with a QIAquick PCR Purification kit (Qiagen, cat#28104) and sequenced at Sequetech (USA) using an Ion Torrent PGM 316 or 318 chip (Life Technologies). Sequencing data were processed by the software package Barracoda (available online at http://www.cbs.dtu.dk/services/barracoda). This tool identifies the DNA barcodes annotated for a given experiment, assigns a sample ID and pMHC specificity to each DNA barcode, and counts the total number of reads and clonally reduced reads for each pMHC-associated DNA barcode. Log_2_ fold changes in read counts mapped to a given sample relative to the mean read counts mapped to triplicate baseline samples are estimated using normalization factors determined by the trimmed mean of M-values method. False discovery rates (FDRs) were estimated using the Benjamini–Hochberg method. At least 1/1,000 reads associated with a given DNA barcode relative to the total number of DNA barcode reads in that given sample was set as threshold to avoid false-positive detection of T cell responses due to low number of reads in the baseline samples. An estimated cell frequency was calculated for each DNA barcode from their read count fraction out of the percentage of CD8^+^ multimer^+^ T cells. DNA barcodes with a *p* < 0.001, which is equal with FDR < 0.1%, and an estimated cell frequency > 0.005%, were considered to be true T cell responses.

### Detection of pMHC-Specific T Cells by Fluorescently Labeled pMHC Tetramers

pMHCs for which T cell responses were detected with the DNA-barcode labeled multimers were generated as fluorescently labeled pMHC tetramers in a combinatorial manner as previously described (42). Briefly, pMHC complexes were multimerized on two different streptavidin-conjugated fluorochromes to give a unique two-color combination. The following streptavidin-conjugated fluorochromes were used: PE (Biolegend, cat#405203), allophycocyanin (APC) (Biolegend, cat#405207), phycoerythrin-cyanin 7 (PE-Cy7) (Biolegend, cat#405206), PE-CF594 (BD, cat#562284), brilliant ultraviolet (BUV)737 (BD, cat#564293), brilliant violet (BV)605 (BD, cat#563260), BV650 (BD, cat#563855), BUV395 (BD, cat#564176), and BV421 (Biolegend, cat#405226). RCC patient TILs were stained with tetramers, followed by a 5× antibody mix composed of CD8-BV510 or -BV480, dump channel antibodies (CD4-FITC, CD14-FITC, CD19-FITC, CD40-FITC, and CD16-FITC), and a dead cell marker (LIVE/DEAD Fixable Near-IR). Multimer positive T cells were gated as single, live, CD8^+^, FITC^−^ (dump channel), multimer color1^+^, multimer color2^+^, and negative for the remaining colors, and defined by a minimum of 10 dual-color positive events.

### Flow Cytometry

All flow cytometry experiments were carried out on LSRFortessa and FACSAria Fusion instruments (BD Biosciences). Data were analyzed in FACSDiva Software version 8.0.2 (BD Biosciences) and FlowJo version 10.4.2 (TreeStar, Inc.).

### Determination of T Cell Diversity

T cell diversity was determined through the identification of CDR3 sequences from bulk RNAseq data with MiXCR version 2.1.1 (43) with the optimized setting for this specific purpose (44). The quality trimmed reads from RNAseq were used as input to MiXCR, which identify specific clones with reference to known CDR3 sequences from the ImMunoGeneTics (IMGT) database. The clone count of each clone detected refers to the reads aligning to this specific clone of the CDR3 reference library. Shannon entropy (45) was calculated as a T cell diversity measurement (46).

### Self-Similarity Score

MuPeXI predicts the corresponding normal peptide for any predicted neopeptide. For a neopeptide derived from SNVs, the most similar normal peptide is identified from the unmutated amino acid sequence in the reference proteome. However, for a neopeptide derived from indels, the reference proteome is searched for the most similar peptide with up to four mismatches, referred to as the nearest normal peptide (32). The self-similarity score between a neopeptide and normal peptides was calculated using the kernel similarity measure (47). In short, this similarity is calculated from matching, at different length scales, all kmers (a substring of length *k*) in one peptide to the kmers in the other peptide using a Blosum similarity measure. The measure gives a value between 0 and 1 for the similarity of two peptides, where a value of 1 indicates a perfect match.

### Statistical Analyses

**Figure 1:**
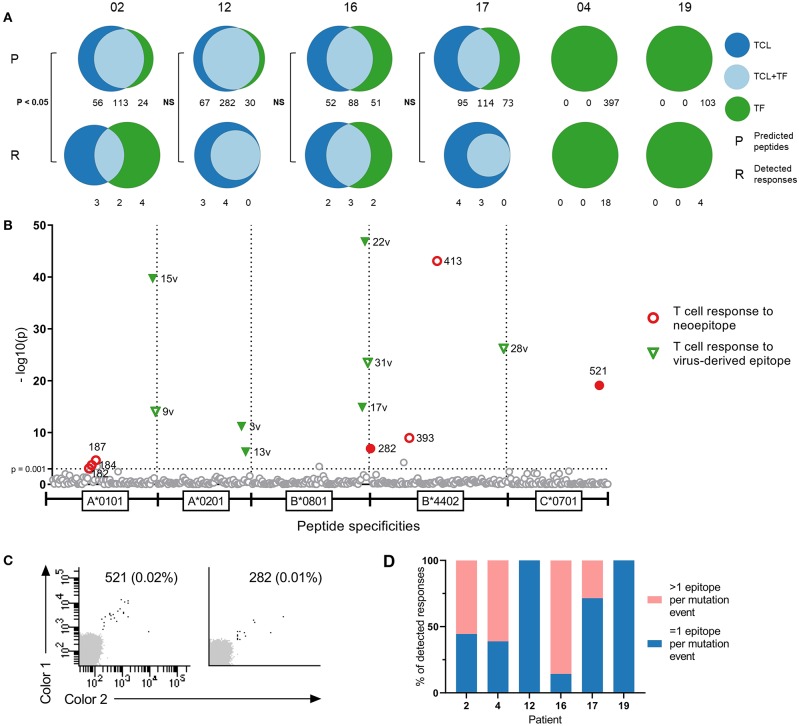
Comparison of tumor cell lines and tumor fragments as sources for neopeptide prediction. **(A)** Distribution of predicted peptides (P) and detected responses (R) across tumor cell lines (TCL), tumor fragments (TF), and tumor cell line-tumor fragment overlap (TCL+TF) in each patient. Distribution of peptides analyzed with Fisher's exact test with Freeman-Halton extension. **(B)** T cell responses in patient RCC12 detected against neoepitopes and virus control epitopes with DNA barcode labeled multimers presented as —log10 of their significance level, distributed on HLA types. Dotted line at x = 3 [—log10(0.001)] represent the selected threshold of FDR < 0.1%. Filled labels indicate responses verified by tetramer staining. **(C)** Examples of tetramer verification plots for two of the responses detected in patient RCC12 against peptide 521 (C*0701) and peptide 282 (B*4402). **(D)** Distribution of responses, where the mutational event gave rise to more or less than 1 epitope in each patient.

**Figure 2:**
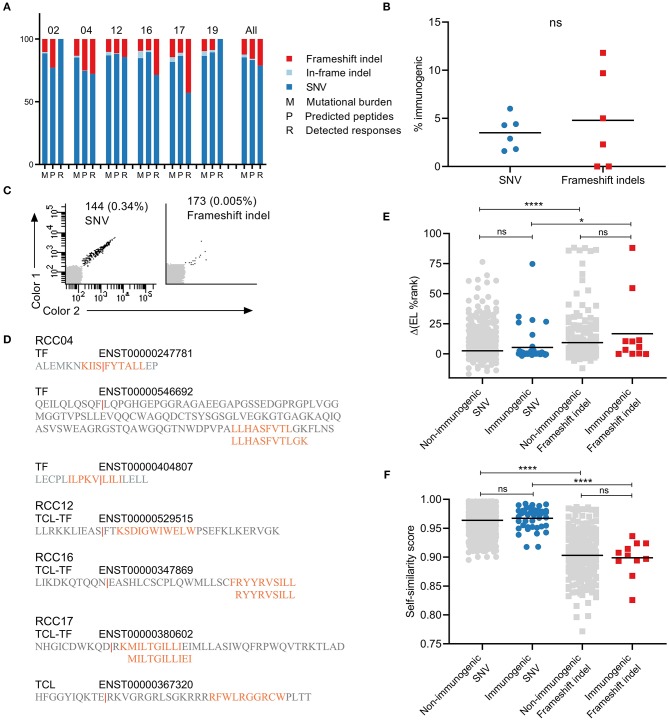
Characterization of the contribution of different mutation types to immunogenicity. **(A)**, Distribution of frameshift indel (red), in-frame indel (light blue), and single nucleotide variation (SNV) (dark blue) mutations in each patient across tumor mutational burden (M), predicted peptides (P), and detected responses (R). **(B)** Percentages of immunogenic neoepitopes out of predicted peptides. NS difference found between mutation types (Mann-Withney U-test). **(C)** Examples of T cell responses detected against SNV mutation (left) and frameshift indel (right) derived neoepitopes. **(D)** Illustration of the frameshift mutational events giving rise to T cell responses in patients RCC04, 12, 16, and 17. **(E)** The difference in % eluted ligand (EL) rank scores between neoepitope and the corresponding wild-type. No difference between non-immunogenic and immunogenic neopeptide within the same mutation type. However, ****p < 0.001 and *p = 0.0315 for comparison between mutation types within the same immunogenicity group (Kruskal-Wallis test with Dunn's correction). **(F)** Self-similarity score between neopeptide and the corresponding wild-type. No difference between non-immunogenic and immunogenic neopeptides within the same mutation type. However, ****p < 0.001 and ****p < 0.001 for comparison between mutation types within the same immunogenicity group (Kruskal-Wallis test with Dunn's correction).

**Figure 3:**
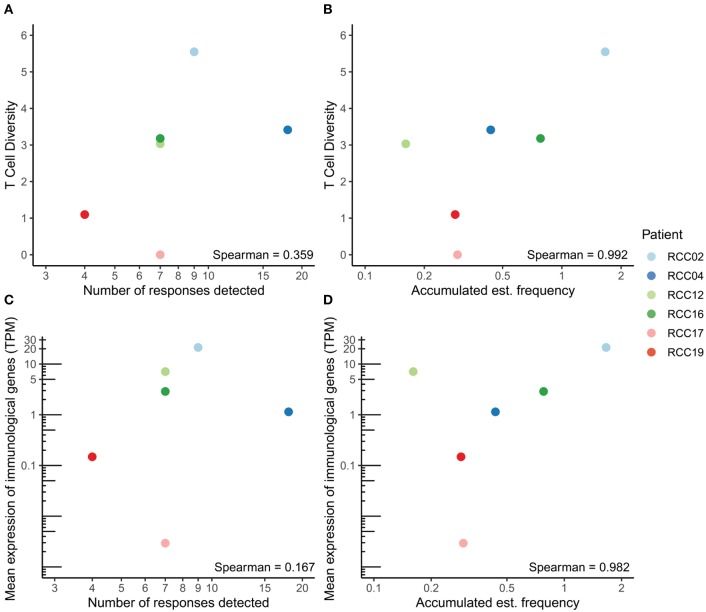
Correlation between T cell diversity, functionality and immunogenicity. **(A,B)** Correlation between the number of detected responses **(A)** or the accumulated estimated frequency **(B)** and T cell diversity in each patient. **(C,D)** Correlation between the number of detected responses **(C)** or the accumulated estimated frequency **(D)** and the mean expression of immunological genes, measured as transcripts per million (TPM). The Spearman correlation coefficient is denoted in each plot.

The difference in the distribution of predicted peptides and detected responses (Figure 1A) was analyzed with Fisher's exact test with the Freeman–Halton extension. The data presented in Figure 2 were assessed for normal distribution with a Shapiro–Wilk normality test with a significance level of 0.05. Data were analyzed with a non-parametric Mann–Whitney *U*-test or Kruskal–Wallis test with Dunn's correction for multiple comparisons. The correlations presented in Figure 3 were analyzed using Spearman's non-parametric correlation. These statistical analyses were conducted using either GraphPad Prism 8.1.2 or R statistically software version 3.5.1.

## Results

### Neopeptides Predicted From Two Sources

The mutational landscape of tumors from four of the six ccRCC patients was analyzed by WES and RNAseq from TFs, autologous TCLs, and TILs (germline reference). For two patients, TCLs were not established, and the analysis was done on TF solely. *In silico* extraction and prediction of neopeptides based on tumor sequencing data was performed with MuPeXI (32). The mutational burden ranged from 51 to 159 mutations in the six patients (Table 1). From these, neopeptides were predicted as 9-, 10-, and 11-mer peptides with predicted binding capacity to the patients' HLAs. Based on available MHC monomers produced in-house, we selected only the HLA types we could cover for the generation of the peptide (p)MHC libraries. Based on this, four to five HLA types per patient were included in the neopeptide prediction. Binders were defined with a predicted rank score below 2 using NetMHCpan 2.8. On average, 258 putative neopeptides were predicted per patient, ranging from 103 to 397 (Table 1).

**Table 1:** Overview of number of mutations, predicted neopeptides, and detected T cell responses for each of the six patients.

	**Mutations**				**Predicted neopeptides**				**Detected responses**			
	**TCL**	**TF**	**TCL/TF**	**Total**	**TCL**	**TF**	**TCL/TF**	**Total**	**TCL**	**TF**	**TCL/TF**	**Total**
RCC02	30	13	43	86	56	24	113	193	3	4	2	9
RCC04	–	138	–	138	–	397	–	397	–	18	–	18
RCC12	28	19	97	144	67	30	282	379	3	0	4	7
RCC16	24	21	38	83	52	51	88	191	2	2	3	7
RCC17	55	39	65	159	95	73	114	282	4	0	3	7
RCC19	–	51	–	51	–	103	–	103	–	4	–	4

*TCL, tumor cell line; TF, tumor fragment*.

In the four patients with two tumor sources available for prediction, we conducted a comparison of the peptide origin. The mutational landscape overlapped substantially with average 50% of mutations detected in both tumor sources, and consequently, more than half of the neopeptides were predicted from both sources (57%, range 40–74%, *n* = 4). However, a proportion of the neopeptides were only predicted from one source: 17% from TF only (range, 8–27%, *n* = 4) and 26% from TCL only (range, 18–34%, *n* = 4). A similar trend is observed in the neoepitopes recognized by T cells (described in detail in the following section) with 40% of the neoepitopes being predicted from both sources (range, 22–57%, *n* = 4), whereas 20% were predicted only from TF (range, 0–44%, *n* = 4) and 40% only from TCL (range, 29–57%, *n* = 4) (Figure 1A). In three of the four patients, there was no difference in the distribution of peptides between predicted peptide and detected responses. However, in patient 02, the distribution was significantly different (Fisher's exact test with Freeman–Halton extension, *p* < 0.05). These results indicate the advantage of applying multiple sources of tumor material for neopeptide prediction to provide a comprehensive identification of T cell responses toward potential neopeptides.

### Neoepitope-Specific CD8^+^ T Cells Are Detected in ccRCC Patients

The 1,545 predicted neopeptides were synthesized and used to generate patient-specific libraries of DNA barcode-labeled pMHC multimers, as previously described (41). Included in each library were HLA matching epitopes derived from common viruses: influenza virus (FLU), Epstein–Barr virus (EBV), and cytomegalovirus (CMV). This resulted in patient-specific library sizes of 114 to 415 pMHC multimers that were used to stain cryopreserved TILs from the corresponding RCC patient and PBMCs from healthy donor controls. All CD8^+^ T cells binding to a given pMHC multimer were selected and sorted based on their positive PE signal. The associated DNA barcodes were amplified and sequenced to reveal the neopeptide specificities recognized within the TIL samples. T cell responses were defined as any pMHC complex enriched in the sorted T cell fraction with a *p* < 0.001 and an estimated cell frequency above 0.005%.

T cell responses toward 54 neopeptides were detected across all patients, ranging from 4 to 18 responses per patient. Figure 1B shows a representation of patient 12. Results from the remaining patients are presented in Figure S1 and with peptide information in Table S1. The recognized neopeptides spanned two to five HLA restrictions and covered, on average, 76% of the HLAs screened for (range, 50–100%). A number of the neopeptides were derived from the same mutational event, resulting in peptides with varying degrees of overlap in sequence. On average, 38% (range, 0–85.7%) of the T cell responses were directed toward mutations where >1 neoepitope was recognized by T cells (Figure 1D). Furthermore, in three of the six patients, T cell responses toward the common virus epitopes were detected (ranging from one to eight responses per patient) (Figure 1B, Figure S1). In the healthy donor cohort, we detected T cell responses toward several epitopes derived from common viruses, as expected. However, low-frequency responses toward neoepitopes were also detected.

The recognized neoepitopes were unique to each patient and none originated from known shared mutations. In a search of the COSMIC database, none of the mutations were previously described in renal cell carcinoma (*n* = 6), and in a broader search of kidney cancer [carcinoma (*n* = 4512), leiomyoblastoma (*n* = 3), renal cell carcinoma (*n* = 6), Wilms tumor (*n* = 1354), not specified (*n* = 106), and other (*n* = 143)], only two mutations were reported with a frequency above 1%: COL14A1 (1%, *n* = 2168) and PCDH11X (2.4%, *n* = 2168).

Fluorescently labeled combinatorial encoding pMHC tetramers were generated for the neoepitopes for which we observed responses with the barcode-labeling method, and these were used to validate the T cell reactivity for a number of the T cell responses observed (filled symbols, Figure 1C and Figure S1). Due to the combinatorial encoding of the tetramers, peptides with great sequence similarity (<2 amino acid difference) were not allowed in the same screen. This was, for instance, the case in patient 16 for neoepitope 144 and 173 with one amino acid difference. Tetramers were only generated for peptide 173, for which we detected T cell response toward, and we, therefore, consider peptide 144 as indirectly validated. In most cases, due to low cell numbers, the cells used for verification screens were from another TIL expanded cell product than the ones used in the original screen, whereby variation might occur—especially as many of the detected responses were of very low frequency. For patient 17 only, a CD107a sorted and expanded cell culture was available, and we screened it with the DNA barcode-labeled multimers. We detected T cell responses toward some of the same neopeptides, as in the original TIL sample (Figure S1).

### Frameshift Indels Contribute to Immunogenicity

The tumor mutational burden of the patients included several non-synonymous mutation types: SNVs, frameshift indels, and in-frame indels (deletions and insertions) (Figure 2A). As expected, SNVs accounted for the largest fraction of mutations in the tumors of all six patients and resulted in a greater number of predicted neopeptides. The two other mutation types are less frequent; on average, across all patients, 12% of mutations and 16% of predicted neopeptides were frameshift indels (range, 9–15 and 9–25%), and 3% of mutations and 1% of predicted neopeptides were in-frame indels (range, 1–6 and 0–2.5%). Only neopeptides derived from SNVs (41/52, 79%) and frameshift indels (11/52, 21%) were recognized by T cells in our screen. There was no significant difference between the percentages of immunogenic neoepitopes out of predicted peptides between the two mutation groups (Figure 2B). However, a slightly increased average fraction was observed for frameshift indels (4.5%, range 0–12%) compared to SNVs (3.2%, range 1–6%). Validation plots of two responses toward each mutation type are presented in Figure 2C. The position of the original mutation that resulted in the frameshift varied between each event. In most cases, the mutation was upstream of the predicted neoepitope, and only a couple of neoepitopes were predicted at the mutation site (Figure 2D).

### Frameshift Indels Have Increased Binding Capacity and Less Similarity to Self

The neoepitope predictor MuPeXI provides the corresponding wild-type peptide for any predicted neopeptide but through different means depending on the mutation type. For a neopeptide derived from SNVs, it is simply the unmutated amino acid sequence in the reference proteome. However, frameshift indels result in an entirely changed amino acid sequence. Instead, the reference proteome is searched for the most similar peptide with up to four mismatches, which will be defined as the nearest normal peptide to the neopeptide (32). In the following, both types will be referred to as wild-type peptides. We first investigated how both mutation types change the MHC binding capacity compared to wild-type. The prediction of neopeptides was performed with NetMHCpan 2.8. However, at the time of publication, a new version was available (NetMHCpan 4.0) (36). Therefore, a second prediction of the current libraries was performed, and the % eluted ligand rank scores from the two versions were compared (Figure S2A). The outputs correlated well, with outliers representing a difference in prediction algorithms between the two versions. We continued with the prediction values from the newest version of NetMHCpan and used it to compare the binding capacity of neopeptides compared to wild-type peptide. The predicted rank scores for neopeptides were generally lower than the wild-type peptides (Figures S2B,C for individual patients). This difference was calculated as a delta(EL %Rank) value and divided into immunogenic and non-immunogenic peptides based on the T cell responses detected with the barcode-labeling method (Figure 2E). Within each mutation group, there was no significant difference between peptides based on their immunogenicity, even though, for both groups, slightly higher average delta values were detected for the immunogenic neoepitopes (SNVs: 2.6 and 5.4; frameshift indels: 9.4 and 16.7 for non-and immunogenic peptides, respectively). Furthermore, between the two mutation types, frameshift mutations had significantly enhanced MHC binding capacity compared to SNVs, relative to their wild-type sequence. Next, we determined the similarity between neopeptide and wild type using the kernel similarity measure giving a score between 0 and 1, where a value of 1 indicates a perfect match (47) (Figure 2F). This approach has previously been shown to focus on the central part of the peptide and could be an indication of similarity in T cell recognition of the presented peptide (48). As before, there is no significant difference within the same mutation group between non-immunogenic and immunogenic neopeptides. However, between the mutation types, neopeptides derived from frameshift indels are significantly less similar to wild type compared to SNV (SNVs: 0.96 and 0.97; frameshift indels: 0.9 and 0.89 for non- and immunogenic peptides, respectively).

### T Cell Diversity and Functionality

We next investigated the T cell tumor infiltration and associated functional markers in the six ccRCC patients. The T cell receptor (TCR) CDR3 sequences were detected from bulk RNAseq data with MiXCR and T cell diversity was calculated using the Shannon Entropy, taking the number of reads per sample into account. Generally, few reads were detected, which is expected when extracting TCR CDR3s from RNAseq data. As a control measure, no TCRs were detected in the TCL samples, except one clone with a single read (data not shown). T cell diversity correlated with both the number of detected responses and accumulated estimated frequency from the DNA barcode screen (Figures 3A,B). The correlation was stronger for the accumulated estimated frequency than for the number of detected responses (Spearman correlation coefficient of 0.992 and 0.359, respectively), indicating T cell diversity as a potential surrogate marker for the number of (neo)antigen-specific T cells in the tumor. We further evaluated CD8 expression and expression of the perforin-granzyme pathway associated with CD8+ T cell activation. The mean expression of these genes correlated with both the number of detected responses and the accumulated estimated frequency from the DNA barcode screen (Figures 3C,D). Again, a strong positive correlation was observed for accumulated estimated frequency, whereas a weak correlation was observed for the number of detected responses (Spearman correlation coefficient of 0.982 and 0.167, respectively), demonstrating that the cell frequencies are better measurements relative to the number of recognized neopeptides.

## Discussion

This study details for the first time the identification and characterization of neoepitopes in renal cell carcinoma. By using a novel, high-throughput technology of DNA barcode-labeled pMHC multimers, we identified a total of 52 neoepitope-specific CD8^+^ T cell responses in TILs from six patients with ccRCC. Renal cell carcinomas are known to harbor the highest number of insertions and deletion of all cancers (ccRCCs scoring highest of renal cell cancer subtypes), and in line with this, mutational analyses revealed the presence of frameshift and in-frame indel mutations in all six patients in the study cohort. Although we detected no responses toward in-frame indels, we observed a tendency of enrichment for T cell responses toward frameshift indel-derived neoepitopes compared to SNV-derived neoepitopes. This supports the notion that indels are a highly immunogenic subgroup of mutations, given their low self-similarity to the wild-type sequence and previous reports of enriched mutant-specific binding. We, therefore, advocate for the inclusion of indel-derived neopeptides in T cell investigational studies and neoepitope-based therapies, also in cancers with low numbers of indels. Although neoepitope prediction pipelines are undergoing intense development and optimization in these years, no consensus exists with respect to the material source for extraction of DNA and RNA for mutational mapping. Our comparison of TFs and TCLs revealed a substantial overlap in the mutational landscape identified based on the two sources (~40% overlap), but none of the source materials performed better than the other in terms of identifying neoepitopes subjected to T cell recognition. Since a large number of epitopes were predicted from only one source or the other, it is advisable (when possible) to include both material sources as input for mutational analyses. A fraction of the variability that we observe between TFs and TCLs might be evenly present between two individual biopsies. Such tumor heterogeneity is well-documented, especially in renal cell carcinoma (49, 50). In the current study, the TFs and TCLs were generated from the same lumps of surgically removed tumors. However, they might still be influenced by tumor heterogeneity.

The neoepitopes detected in this study are all MHC class I restricted. Within recent years, growing interest have been on MHC class II neoantigens and the important role of CD4^+^ T cells in tumor recognition and in generating a strong anti-tumor response (51, 52). Several cancer vaccines have shown to generate immune responses to class II neoepitopes either alone or in combination with class I neoepitopes (9, 53). CD4^+^ T cells have also been suggested to be critical for tumor regression during checkpoint inhibitor therapy (54). Still, limitations in both *in silico* prediction algorithms and MHC-II multimer staining reagents make identification of neoepitope-specific CD4^+^ T cells challenging (55).

Although the number of RCC patients evaluated in this study is limited, the neoepitope screening presented here covers 1,545 predicted neoepitopes, derived from 572 SNV mutations and 99 frameshift/indel mutations, with ligands binding to 16 different HLA class I molecules. Thus, despite the limited number of patients analyzed, this represents a broad screening effort of class I neoepitopes from both SNVs and frameshift mutations, providing new insight into the neoepitope landscape in renal cell carcinoma patients. In line with previous studies of neoepitopes in other cancer types, all of the neoepitopes derived from mutations were unique to the given patient. Thus, therapeutic utilization in precision-targeted approaches will require patient-specific mutational mapping and prediction of neoepitopes, which can then be applied to tailor-made therapies such as personalized cancer vaccines or adoptive transfer of expanded neoepitope-specific patient TILs. The identification of virus-specific bystander T cells in the TIL products of half of the patients document the presence of therapeutically irrelevant T cells in current treatment products and further supports the rationale of developing precision-targeted therapies.

## Data Availability Statement

The raw data supporting the conclusions of this article will be made available by the authors, without undue reservation, to any qualified researcher.

## Ethics Statement

The studies involving human participants were reviewed and approved by the Ethics Committee of the Capital Region of Denmark. The Danish Data Protection Agency. The patients/participants provided their written informed consent to participate in this study.

## Author Contributions

UH and SR designed and performed experiments, data analysis, generated figures, and wrote the manuscript. A-MB designed the *in silico* prediction platform, performed data analysis, and generated figures. AB performed data analysis, generated figures, and revised the manuscript. RA provided donor material, diagnosed and characterized the patients, and generated tumor cell lines. AD, MD, and IS provided donor material and generated tumor cell lines. AKB and AM provided technical assistance, discussed data, and revised the manuscript. ZS and AE designed the *in silico* platforms. SH conceived the concept, supervised the study, discussed the data, and wrote the manuscript.

### Conflict of Interest

AE was employed by the company Clinical Microbiomics A/S. The remaining authors declare that the research was conducted in the absence of any commercial or financial relationships that could be construed as a potential conflict of interest.
