# Supplementary figures and images for: Tumor-Infiltrating T Cells From Clear Cell Renal Cell Carcinoma Patients Recognize Neoepitopes Derived From Point and Frameshift Mutations
Source: Front Immunol. 2020 Mar 12;11:373. doi: 10.3389/fimmu.2020.00373 (PMC7080703; doi:10.3389/fimmu.2020.00373)

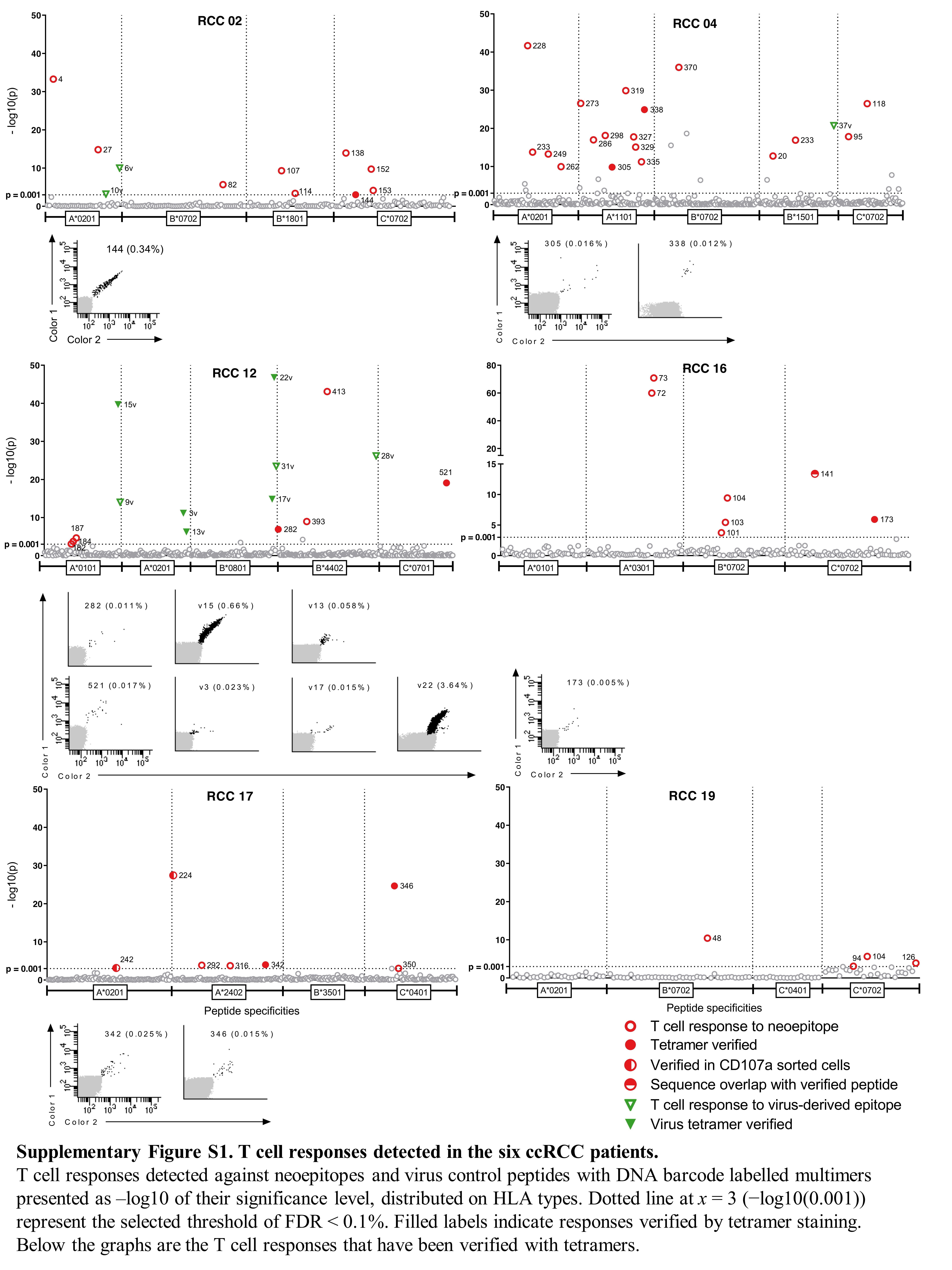

Supplement: Supplementary file 1 [file Image_1.jpg]

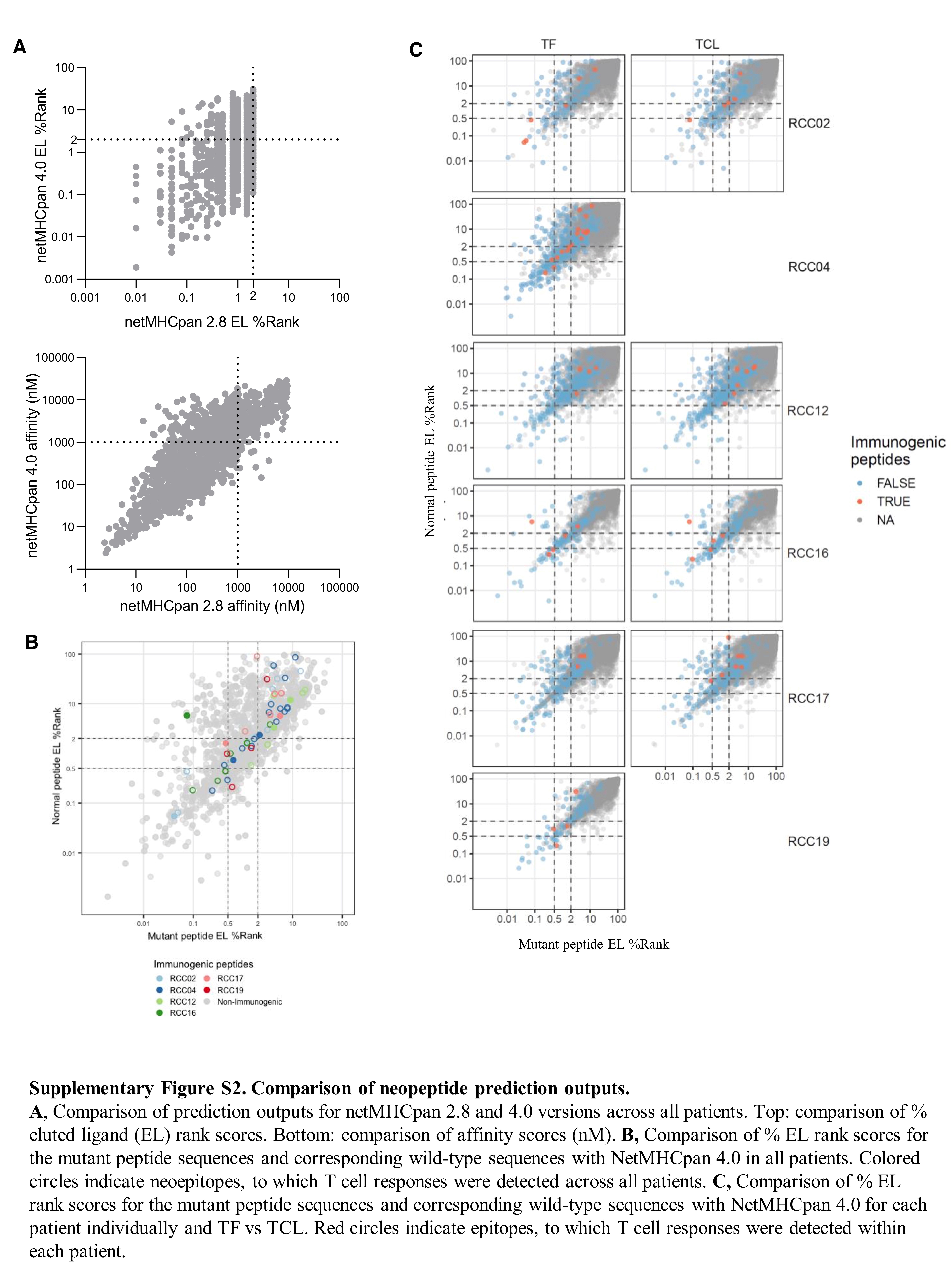

Supplement: Supplementary file 2 [file Image_2.jpg]
