# Supplementary material for: Tumor-Infiltrating T Cells From Clear Cell Renal Cell Carcinoma Patients Recognize Neoepitopes Derived From Point and Frameshift Mutations
Source: Front Immunol. 2020 Mar 12;11:373. doi: 10.3389/fimmu.2020.00373 (PMC7080703; doi:10.3389/fimmu.2020.00373)
Supplement: Supplementary file 3 [file Table_1.docx]

**Supplementary Table S1:** List of T cell epitopes detected

| Patient ID | Peptide ID | HLA | WT sequence | WT rank score (netMHCpan 2.8) | WT rank score (netMHCpan 4.0) | WT Affinity (netMHCpan 2.8) | Mut sequence | Mut rank score (netMHCpan 2.8) | Mut rank score (netMHCpan 4.0) | Mut Affinity (netMHCpan 2.8) | Gene ID | Transcript ID | Amino Acid Change | Mismatches | Peptide position | Chromosome | Genomic Position | Protein position | Mutation Consequence | HUGO Symbol | Cancer Driver Gene | Mut affinity score | WT affinity score | Expression (RPM) | Prioritization Score | Origin |
| --- | --- | --- | --- | --- | --- | --- | --- | --- | --- | --- | --- | --- | --- | --- | --- | --- | --- | --- | --- | --- | --- | --- | --- | --- | --- | --- |
| 02 | 4 | A*0201 | SLLRFLYTQL | 1 | 2.16 | 22.97 | FLLRFLYTQL | 0.15 | 1.86 | 5.26 | ENSG00000197381 | ENST00000389861 | S/F | 1 | 1 | 21 | 45182711 | 430 | M | ADARB1 | No | 0.999904 | 0.993307 | 8.8 | 5 | TCL |
|  | 27 | A*0201 | TLIAIFPYAGL | 1.5 | 2.95 | 34.95 | TLIAIFPYARL | 2 | 3.10 | 55.74 | ENSG00000125454 | ENST00000416858 | G/R | 1 | 10 | 17 | 75278227 | 190 | M | SLC25A19 | No | 0.5 | 0.924142 | 4.8 | 3 | TCL |
|  | 82 | B*0702 | SPPRPGRLPDL | 0.8 | 1.68 | 122.24 | SPPHPGRLPDL | 0.8 | 1.36 | 108.21 | ENSG00000136099 | ENST00000338862 | R/H | 1 | 4 | 13 | 52844663 | 940 | M | PCDH8 | No | 0.997527 | 0.997527 | 0 | 5 | TCL/TF |
|  | 107 | B*1801 | KENSLITQF | 0.8 | 0.06 | 499.47 | KENSLIIQF | 0.5 | 0.05 | 237.63 | ENSG00000102699 | ENST00000381989 | T/I | 1 | 7 | 13 | 24442624 | 1170 | M | PARP4 | No | 0.999447 | 0.997527 | 35.8 | 5 | TF |
|  | 114 | B*1801 | MEAIWLYQF | 0.05 | 0.05 | 11.82 | MEAIRLYQF | 0.15 | 0.04 | 47.85 | ENSG00000155961 | ENST00000369454 | W/R | 1 | 5 | X | 155264276 | 5 | M | RAB39B | No | 0.999904 | 0.999942 | 0.84 | 5 | TF |
|  | 138 | C*0702 | AGLQFSCYSSL | 32 | 31.61 | 11632.1 | ARLQFSCYSSL | 0.8 | 4.90 | 284.85 | ENSG00000125454 | ENST00000416858 | G/R | 1 | 2 | 17 | 75278227 | 190 | M | SLC25A19 | No | 0.997527 | 0.000000 | 4.8 | 10 | TCL |
|  | 144 | C*0702 | FRQKTNLIV | 0.4 | 0.43 | 176 | FRQKTNLIL | 0.08 | 0.07 | 46.98 | ENSG00000189042 | ENST00000588311 | V/L | 1 | 9 | 19 | 36720540 | 575 | M | ZNF567 | No | 0.999932 | 0.999665 | 5.1 | 5 | TCL/TF |
|  | 152 | C*0702 | IWLYQFRLIV | 15 | 19.77 | 9265.8 | IRLYQFRLIV | 2 | 3.94 | 961.3 | ENSG00000155961 | ENST00000369454 | W/R | 1 | 2 | X | 155264276 | 5 | M | RAB39B | No | 0.500000 | 0.000000 | 0.84 | 5 | TF |
|  | 153 | C*0702 | IWLYQFRLIVI | 15 | 45.39 | 8974.4 | IRLYQFRLIVI | 2 | 14.48 | 990.78 | ENSG00000155961 | ENST00000369454 | W/R | 1 | 2 | X | 155264276 | 5 | M | RAB39B | No | 0.500000 | 0.000000 | 0.84 | 5 | TF |
| 04 | 20 | B*1501 | VLICLRLLMRD | 50 | 86.00 | 29264.9 | VLICLRLLMRY | 2 | 11.28 | 278.49 | ENSG00000163491 | ENST00000429845 | D/Y | 1 | 11 | 3 | 27322191 | 145 | M | NEK10 | No | 0.500000 | 0.000000 | 6.7 | 5 | TF |
|  | 95 | C*0702 | FRTIYLNTLL | 0.3 | 1.26 | 126.6 | FRSIYLNTLL | 0.15 | 0.97 | 74.32 | ENSG00000174080 | ENST00000310325 | T/S | 1 | 3 | 11 | 66566152 | 246 | M | CTSF | No | 0.999904 | 0.999797 | 25.9 | 5 | TF |
|  | 118 | C*0702 | LYLWFKTACL | 1.5 | 7.98 | 615.3 | LYIWFKTACL | 1 | 5.65 | 471.48 | ENSG00000114302 | ENST00000437821 | L/I | 1 | 3 | 3 | 48751327 | 187 | M | PRKAR2A | No | 0.993307 | 0.924142 | 9.8 | 5 | TF |
|  | 228 | A*0201 | GIINFYTAL | 4 | 6.70 | 250.3 | KIISFYTALL | 1.5 | 3.34 | 41.01 | ENSG00000187955 | ENST00000297848 | F/X | 3 | 05:10 | 8 | 120270121 | 1054 | F | COL14A1 | No | 0.924142 | 0.000045 | 3.3 | 9 | TF |
|  | 233 | A*0201 | LLAASFETL | 1 | 0.29 | 22.7 | LLHASFVTL | 1.5 | 0.49 | 51.57 | ENSG00000166578 | ENST00000546692 | Y/LX | 2 | 01:09 | 12 | 113200626-113200627 | 306 | F | IQCD | No | 0.924142 | 0.993307 | 1.5 | 7 | TF |
|  | 233 | B*1501 | LLAASFETL | 1 | 1.95 | 22.7 | LLHASFVTL | 1.5 | 1.69 | 51.57 | ENSG00000166578 | ENST00000546692 | Y/LX | 2 | 01:09 | 12 | 113200626-113200627 | 306 | F | IQCD | No | 0.924142 | 0.993307 | 1.5 | 7 | TF |
|  | 249 | A*0201 | MLKYQKILWKV | 3 | 7.99 | 144.6 | MLEYQKILWKV | 2 | 7.85 | 85.99 | ENSG00000088756 | ENST00000419673 | K/E | 1 | 3 | 18 | 6890062 | 412 | M | ARHGAP28 | No | 0.500000 | 0.006693 | 2.1 | 5 | TF |
|  | 262 | A*0201 | FLPKVLDLI | 0.8 | 0.18 | 14.8 | ILPKVLILI | 2 | 0.24 | 72.43 | ENSG00000204406 | ENST00000407073 | F/X | 2 | 06:09 | 2 | 148463742 | 74 | F | MBD5 | No | 0.500000 | 0.997527 | 29.4 | 4 | TF |
|  | 273 | A*1101 | TQFLYLWFK | 0.05 | 0.58 | 9.05 | TQFLYIWFK | 0.08 | 0.42 | 10.52 | ENSG00000114302 | ENST00000437821 | L/I | 1 | 6 | 3 | 48751327 | 187 | M | PRKAR2A | No | 0.999932 | 0.999942 | 9.8 | 5 | TF |
|  | 286 | A*1101 | LSPACSVTLGK | 1.5 | 58.75 | 135.6 | LLHASFVTLGK | 0.5 | 4.13 | 38.54 | ENSG00000166578 | ENST00000546692 | Y/LX | 4 | 01:11 | 12 | 113200626-113200627 | 306 | F | IQCD | No | 0.999447 | 0.924142 | 1.5 | 9 | TF |
|  | 298 | A*1101 | ALFLVFGRTKK | 0.8 | 1.41 | 45.3 | ALFLVFGRTRK | 0.8 | 1.50 | 45.3 | ENSG00000125965 | ENST00000374372 | K/R | 1 | 10 | 20 | 35434369 | 349 | M | GDF5 | No | 0.997527 | 0.997527 | 0.1 | 5 | TF |
|  | 305 | A*1101 | HIIRLMLKY | 1.5 | 0.73 | 132.5 | HIIRLMLEY | 1 | 0.64 | 88.75 | ENSG00000088756 | ENST00000419673 | K/E | 1 | 8 | 18 | 6890062 | 412 | M | ARHGAP28 | No | 0.993307 | 0.924142 | 2.1 | 5 | TF |
|  | 319 | A*1101 | GTCVRVTVQAR | 4 | 9.76 | 944.2 | GTYVRVTVQAR | 1.5 | 3.69 | 142.52 | ENSG00000101220 | ENST00000217195 | C/Y | 1 | 3 | 20 | 3754429 | 156 | M | C20orf27 | No | 0.924142 | 0.000045 | 6.5 | 9 | TF |
|  | 327 | A*1101 | LMLKYQKILWK | 1.5 | 8.41 | 155.01 | LMLEYQKILWK | 1.5 | 7.80 | 130.13 | ENSG00000088756 | ENST00000419673 | K/E | 1 | 4 | 18 | 6890062 | 412 | M | ARHGAP28 | No | 0.924142 | 0.924142 | 2.1 | 5 | TF |
|  | 329 | A*1101 | WTGATITVKIK | 3 | 6.94 | 568.5 | WTGATITVTIK | 2 | 6.90 | 200.29 | ENSG00000164220 | ENST00000296641 | K/T | 1 | 9 | 5 | 76618507 | 67 | M | F2RL2 | No | 0.500000 | 0.006693 | 0.3 | 5 | TF |
|  | 335 | A*1101 | HTLLLREHNR | 3 | 4.35 | 433.6 | HTLLLWEHNR | 2 | 4.72 | 240.73 | ENSG00000005381 | ENST00000225275 | R/W | 1 | 6 | 17 | 58275646 | 421 | M | MPO | No | 0.500000 | 0.006693 | 0.3 | 5 | TF |
|  | 338 | A*1101 | MLKYQKILWK | 2 | 2.34 | 250.2 | MLEYQKILWK | 2 | 2.16 | 237.43 | ENSG00000088756 | ENST00000419673 | K/E | 1 | 3 | 18 | 6890062 | 412 | M | ARHGAP28 | No | 0.500000 | 0.500000 | 2.1 | 4 | TF |
|  | 370 | B*0702 | CVRVTVQARVM | 1.5 | 33.10 | 368.3 | YVRVTVQARVM | 0.5 | 7.03 | 66.35 | ENSG00000101220 | ENST00000217195 | C/Y | 1 | 1 | 20 | 3754429 | 156 | M | C20orf27 | No | 0.999447 | 0.924142 | 6.2 | 5 | TF |
| 12 | 182 | A*0101 | ITSQSLRSPMY | 0.17 | 0.58 | 150.4 | ITCQSLRSPMY | 0.8 | 1.46 | 959.41 | ENSG00000181903 | ENST00000314259 | S/C | 1 | 3 | 11 | 55665314 | 50 | M | OR4C6 | No | 0.997527 | 0.999894 | 0 | 5 | TCL |
|  | 184 | A*0101 | ITVPRCMKMAY | 0.8 | 1.51 | 1158.9 | ITVARCMKMAY | 0.8 | 3.13 | 977.56 | ENSG00000164930 | ENST00000522484 | P/A | 1 | 4 | 8 | 103300195 | 30 | M | FZD6 | No | 0.997527 | 0.997527 | 40.1 | 5 | TCL/TF |
|  | 187 | A*0101 | SSDIGWVLELP | 4 | 14.70 | 7495.7 | KSDIGWIWELW | 2 | 4.14 | 3455.7 | ENSG00000197302 | ENST00000316491 | FQKVI/FX | 4 | 01:11 | 16 | 31753824-31753834 | 95 | F | ZNF720 | No | 0.500000 | 0.000045 | 8.1 | 5 | TCL/TF |
|  | 282 | B*4402 | YMMNSVLENF | 1.5 | 11.87 | 34.7 | YMMNSVQENF | 2 | 9.06 | 92.27 | ENSG00000074219 | ENST00000593945 | L/Q | 1 | 7 | 19 | 49342460 | 407 | M | TEAD2 | No | 0.500000 | 0.924142 | 13.4 | 3 | TCL/TF |
|  | 393 | B*4402 | IQIFTIVLVSY | 4 | 19.07 | 5498 | IQMFTIVLVSY | 2 | 17.85 | 2030.55 | ENSG00000206536 | ENST00000383695 | I/M | 1 | 3 | 3 | 98391289 | 208 | M | OR5K3 | No | 0.500000 | 0.000045 | 0 | 5 | TCL |
|  | 413 | B*4402 | METLHIIYSEA | 1.5 | 16.55 | 1637.2 | METLHIIYLEA | 1.5 | 15.91 | 1127.35 | ENSG00000180667 | ENST00000367084 | S/L | 1 | 9 | 1 | 207052210 | 9 | M | YOD1 | No | 0.924142 | 0.924142 | 2.0 | 5 | TCL/TF |
|  | 521 | C*0701 | VRRASGCLITL | 0.2 | 3.29 | 192.4 | VRRASVCLITL | 0.17 | 4.24 | 160.75 | ENSG00000049167 | ENST00000265038 | G/V | 1 | 6 | 5 | 60899683 | 221 | M | ERCC8 | No | 0.999894 | 0.999877 | 5.8 | 5 | TCL |
| 16 | 72 | A*0301 | RVYKCLFNH | 0.4 | 1.00 | 65.3 | RVYKCIFNH | 0.3 | 0.56 | 55.38 | ENSG00000090863 | ENST00000205061 | L/I | 1 | 6 | 16 | 74494798 | 338 | M | GLG1 | No | 0.999797 | 0.999665 | 77.7 | 5 | TCL |
|  | 73 | A*0301 | RVYKCLFNHK | 0.01 | 0.18 | 9.3 | RVYKCIFNHK | 0.03 | 0.10 | 9.49 | ENSG00000090863 | ENST00000205061 | L/I | 1 | 6 | 16 | 74494798 | 338 | M | GLG1 | No | 0.999947 | 0.999952 | 77.7 | 5 | TCL |
|  | 101 | B*0702 | LPALAFAFFSL | 0.17 | 1.62 | 17.5 | LPALAFTFFSL | 0.15 | 1.21 | 15.56 | ENSG00000277893 | ENST00000622030 | A/T | 1 | 7 | 2 | 31529356 | 217 | M | SRD5A2 | No | 0.999904 | 0.999894 | 0 | 5 | TCL/TF |
|  | 103 | B*0702 | VPLVILIVL | 2 | 0.28 | 846.4 | MPLVILIVL | 1 | 0.31 | 205.83 | ENSG00000158481 | ENST00000368170 | V/M | 1 | 1 | 1 | 158293253 | 311 | M | CD1C | No | 0.993307 | 0.500000 | 0.2 | 7 | TF |
|  | 104 | B*0702 | VPLVILIVLVL | 1.5 | 3.83 | 462.8 | MPLVILIVLVL | 0.8 | 3.50 | 122.61 | ENSG00000158481 | ENST00000368170 | V/M | 1 | 1 | 1 | 158293253 | 311 | M | CD1C | No | 0.997527 | 0.924142 | 0.2 | 5 | TF |
|  | 141 | C*0702 | FRIYAVSTLL | 0.05 | 0.44 | 38.9 | FRYYRVSILL | 0.01 | 0.44 | 14.53 | ENSG00000003756 | ENST00000347869 | R/X | 3 | 01:10 | 3 | 50105674 | 274 | F | RBM5 | No | 0.999952 | 0.999942 | 39.7 | 9 | TCL/TF |
|  | 173 | C*0702 | NPRRVSILL | 32 | 5.80 | 16100.6 | RYYRVSILL | 0.4 | 0.07 | 146.3 | ENSG00000003756 | ENST00000347869 | R/X | 3 | 01:09 | 3 | 50105674 | 274 | F | RBM5 | No | 0.999665 | 0.000000 | 39.7 | 10 | TCL/TF |
| 17 | 224 | A*2402 | KMLLTEILL | 3 | 16.27 | 105.1 | KMILTGILLI | 2 | 5.92 | 70.32 | ENSG00000198759 | ENST00000361306 | R/X | 3 | 01:10 | X | 13623885 | 415 | F | EGFL6 | No | 0.500000 | 0.006693 | 0 | 5 | TCL/TF |
|  | 242 | A*0201 | MINTSILLIFI | 2 | 15.74 | 88.8 | MILTGILLIEI | 1.5 | 4.36 | 29.07 | ENSG00000198759 | ENST00000361306 | R/X | 3 | 01:11 | X | 13623885 | 415 | F | EGFL6 | No | 0.924142 | 0.500000 | 0 | 9 | TCL/TF |
|  | 292 | A*2402 | QFLESCDEVI | 3 | 6.01 | 1714.04 | KFLESCDEVI | 1 | 3.55 | 384.29 | ENSG00000140798 | ENST00000311303 | Q/K | 1 | 1 | 16 | 48115417 | 663 | M | ABCC12 | No | 0.993307 | 0.006693 | 0 | 10 | TCL/TF |
|  | 316 | A*2402 | SAWLPGGRCS | 50 | 90.00 | 43577.8 | RFWLRGGRCW | 1.5 | 1.93 | 724.85 | ENSG00000118194 | ENST00000509001 | R/X | 4 | 01:10 | 1 | 201362014 | 196 | F | TNNT2 | No | 0.924142 | 0.000000 | 0.1 | 9 | TCL |
|  | 342 | A*2402 | VFTQSFVTVSI | 0.8 | 5.70 | 259.7 | VFTQSFITVSI | 1 | 5.59 | 330.56 | ENSG00000102290 | ENST00000406881 | V/I | 1 | 7 | X | 91877651 | 471 | M | PCDH11X | No | 0.993307 | 0.997527 | 0.1 | 5 | TCL |
|  | 346 | C*0401 | YFATLITELV | 2 | 1.60 | 1139.9 | YFATLITELF | 0.12 | 0.45 | 21.13 | ENSG00000143105 | ENST00000369771 | V/F | 1 | 10 | 1 | 110517818 | 324 | M | KCNA10 | No | 0.999917 | 0.500000 | 0 | 7 | TCL |
|  | 350 | C*0401 | YLCAGAVDF | 4 | 2.79 | 2987.2 | YLCAGAVYF | 2 | 1.11 | 1194.53 | ENSG00000104812 | ENST00000323798 | D/Y | 1 | 8 | 19 | 48985873 | 219 | M | GYS1 | No | 0.500000 | 0.000045 | 32.9 | 5 | TCL |
| 19 | 48 | B*0702 | IPDSIAVIQQL | 1.5 | 1.29 | 549.9 | IPDSIALIQQL | 1.5 | 1.47 | 403.1 | ENSG00000205413 | ENST00000379958 | V/L | 1 | 7 | 7 | 93103541 | 853 | M | SAMD9 | No | 0.924142 | 0.924142 | 0.2 | 5 | TF |
|  | 94 | C*0702 | NGHDCWSLISL | 15 | 31.20 | 19831.8 | NRHDCWSLISL | 1.5 | 3.05 | 7886.91 | ENSG00000174016 | ENST00000538312 | G/R | 1 | 2 | X | 80442957 | 140 | M | FAM46D | No | 0.924142 | 0.000000 | 0 | 9 | TF |
|  | 104 | C*0702 | GRQSWSSYV | 1.5 | 0.98 | 583.1 | GRQSWSLYV | 0.4 | 0.48 | 181.15 | ENSG00000137210 | ENST00000473276 | S/L | 1 | 7 | 6 | 10756495 | 48 | M | TMEM14B | No | 0.999665 | 0.924142 | 1.1 | 5 | TF |
|  | 126 | C*0702 | FWYYAKVEL | 0.8 | 0.21 | 246.7 | FWYYAKVDL | 2 | 0.61 | 965.39 | ENSG00000167283 | ENST00000524422 | E/D | 1 | 8 | 11 | 118406995 | 37 | M | ATP5L | No | 0.500000 | 0.997527 | 3.4 | 2 | TF |

RCC, renal cell carcinoma, WT: wild type, Mut: mutant, HUGO: human genome organization, RPM: reads per million.
